# Open-Label Placebo for the Treatment of Cancer-Related Fatigue in Patients with Advanced Cancer: A Randomized Controlled Trial

**DOI:** 10.1093/oncolo/oyac184

**Published:** 2022-09-15

**Authors:** Sriram Yennurajalingam, Ahsan Azhar, Zhanni Lu, Ashley J Rodriguez, Adrienne B Arechiga, Maria Guerra-Sanchez, Penny Stanton, Clark R Andersen, Diana L Urbauer, Eduardo Bruera

**Affiliations:** The University of Texas MD Anderson Cancer Center, Department of Palliative Care, Rehabilitation Medicine, and Integrative Medicine, Houston, TX, USA; The University of Texas MD Anderson Cancer Center, Department of Palliative Care, Rehabilitation Medicine, and Integrative Medicine, Houston, TX, USA; The University of Texas MD Anderson Cancer Center, Department of Palliative Care, Rehabilitation Medicine, and Integrative Medicine, Houston, TX, USA; The University of Texas MD Anderson Cancer Center, Department of Palliative Care, Rehabilitation Medicine, and Integrative Medicine, Houston, TX, USA; The University of Texas MD Anderson Cancer Center, Department of Palliative Care, Rehabilitation Medicine, and Integrative Medicine, Houston, TX, USA; The University of Texas MD Anderson Cancer Center, Department of Palliative Care, Rehabilitation Medicine, and Integrative Medicine, Houston, TX, USA; The University of Texas MD Anderson Cancer Center, Department of Palliative Care, Rehabilitation Medicine, and Integrative Medicine, Houston, TX, USA; The University of Texas MD Anderson Cancer Center, Biostatistics, Houston, TX, USA; The University of Texas MD Anderson Cancer Center, Biostatistics, Houston, TX, USA; The University of Texas MD Anderson Cancer Center, Department of Palliative Care, Rehabilitation Medicine, and Integrative Medicine, Houston, TX, USA

**Keywords:** cancer-related fatigue, treatment, placebo, fatigue cluster, randomized control trial

## Abstract

**Background:**

The purpose of this study was to determine the effects of an open-labeled placebo (OLP) compared to a waitlist control (WL) in reducing cancer-related fatigue (CRF) in patients with advanced cancer using Functional Assessment of Chronic Illness Therapy-Fatigue (FACIT-F).

**Materials and Methods:**

In this randomized controlled trial, patients with fatigue ≥4/10 on Edmonton Symptom Assessment Scale (ESAS) were randomized to OLP one tablet twice a day or WL for 7 days. On day 8, patients of both arms received a placebo for 3 weeks. Changes in FACIT-F from baseline to day 8 (primary outcome) and at day 29, were assessed. Secondary outcomes included FACT-G, Multidimensional Fatigue Symptom Inventory-SF, Fatigue cluster (defined as a composite of ESAS fatigue, pain, and depression), Center for epidemiologic studies-depression, Godin leisure-time physical activity questionnaire, and global symptom evaluation.

**Results:**

A total of 84/90 (93%) patients were evaluable. The mean (SD) FACIT-F change at day 8 was 6.6 (7.6) after OLP, vs. 2.1 (9.4) after WL (*P* = .016). On days 15 and 29, when all patients received OLP, there was a significant improvement in CRF and no difference between arms. There was also a significant improvement in ESAS fatigue, and fatigue cluster score in the OLP arm on day 8 of the study (0.029, and 0.044, respectively). There were no significant differences in other secondary outcomes and adverse events between groups.

**Conclusions:**

Open-labeled placebo was efficacious in reducing CRF and fatigue clusters in fatigued advanced cancer patients at the end of 1 week. The improvement in fatigue was maintained for 4 weeks. Further studies are needed.

## Teaser

This article reports on the effects of an open-labeled placebo compared with wait-list control in reducing cancer-related fatigue in patients with advanced cancer using Functional Assessment of Chronic Illness Therapy-Fatigue (FACIT-F).

**Implications for Practice**

Open-labeled placebo (OLP) was efficacious in reducing cancer-related fatigue (CRF), and fatigue cluster in advanced cancer patients, and the improvement in CRF was maintained for up to 4 weeks. We anticipate that this information will assist cancer researchers in potentially developing clinical trials for the treatment of CRF in advanced cancer using a placebo more as an adjuvant or dose extender, rather than as a control medication. This information also will assist clinicians to interpret the results of the published CRF trials more closely due to the placebo’s role in CRF improvement.

## Introduction

Cancer-related fatigue (CRF) is the most common and debilitating symptom in patients with advanced cancer and is estimated to be present in 60%-90% of these patients.^1-3^ Several randomized controlled trials (RCT) using pharmacological agents for the treatment of CRF were conducted in patients with advanced cancer including the use of erythropoietic stimulating agents, donepezil, corticosteroids, panax ginseng, and psychostimulants such as methylphenidate.^4-8^ The results of these RCTs showed that these agents were often not significantly better compared to the placebo (methylphenidate, donepezil, panax ginseng),^7-10^ or had side effects that prevent their long-term use (erythropoietin, dexamethasone).^4,7,9^ One important reason for the negative studies was that the “placebo effect” may have negatively impacted these RCTs.^7-12^ The placebo effect has been defined as “a beneficial effect, produced by a placebo drug or treatment, that cannot be attributed to the properties of the placebo itself, and must therefore be due to the patient’s belief in that treatment.”^13^ Placebo is known to significantly contribute to the responses in the symptom control clinical trials.^12^ Recent studies have found that a placebo given without deception (open-labeled placebo or OLP) was found to be effective in reducing symptoms in patients with chronic pain, episodic migraine attacks, allergic rhinitis, major depression, menopausal hot flashes, attention deficit hyperactivity disorder, irritable bowel syndrome, and in cancer survivors with CRF.^14-21^ OLP studies found that the symptoms were significantly improved compared to usual clinical care without any adverse effects due to OLP. The open-label format of placebo use was also found to be an acceptable modality of treatment thereby potentially improving the chance of using placebo in routine clinical practice without risking ethical aspects of clinical care, and patient-physician relationship.

Prior studies evaluating the placebo effect in oncology trials found that placebos were sometimes associated with improved control of symptoms such as cancer-related fatigue, pain, and appetite, whereas improvement of objective measures of weight gain and tumor response were rarely observed in the placebo group.^12,22^ Our group investigated previously the placebo effect in CRF clinical studies in patients with advanced cancer.^23,24^ We found that 56% of patients had a placebo response.^23,24^ However, no randomized controlled trials are using OLP and waitlist control for the treatment of CRF focused on patients with advanced cancer, in whom CRF is more frequent and severe than in cancer survivors.^1-3^ Our primary aim was to determine the effects of OLP compared to waitlist control (WL) for reducing CRF in patients with advanced cancer as measured by the Functional Assessment of Chronic Illness Therapy-Fatigue (FACIT-F) at the end of 1 week. We hypothesized that OLP was effective in reducing CRF among patients with advanced cancer. The secondary aims of this study were to examine the effect of OLP and WL on Quality of Life (Functional Assessment of Cancer Illness Therapy-General, FACT-G), various fatigue dimensions—(Multidimensional Fatigue Symptom Inventory, MFSI-SF), cancer symptoms and symptom distress (Edmonton Symptom Assessment Scale, ESAS), Fatigue symptom cluster (ESAS fatigue, pain, and depression), depression (The Center for Epidemiologic Studies—Depression, CES-D), Godin leisure-time physical activity questionnaire (Godin), and global symptom evaluation (GSE), at the days 8 and 29 in both groups. We also examined the study medication adherence and its safety.

## Methods

Site, ethics statement, and trial registration: Patients were enrolled in the outpatient supportive care, and oncology clinics at The University of Texas M.D Anderson Cancer (UT MDACC). The institutional review board of the UT MDACC approved this protocol, and all participants signed an informed consent as a condition of enrollment in the trial. The study was activated for patient accrual on June 21, 2019, and was closed to new patient accrual on May 25, 2021. Trial Registration: NCT03927885.

### Participants

Patients' eligibility criteria included: (a) diagnosis of advanced cancer (metastatic or recurrent incurable solid tumors) with the average intensity of fatigue of ≥4/10 on the Edmonton Symptom Assessment Scale (ESAS)^25^ in the previous 24 h, (b) presence of fatigue for a duration of at least 2 weeks, (c) normal cognition, (d) hemoglobin ≥8 g/L within 1 week of enrollment, and (e) patients were excluded if they had a history of substance abuse (CAGE ≥2+), any surgery in the past month, or uncontrolled pain.

### Treatment and Study Procedure

#### Study Design

Using a waitlist control crossover design, participants were initially randomized to OLP or waitlist crossover WL groups for 7 days (randomized controlled phase). During this phase patients in the OLP group received 1 tablet of placebo twice a day on an open-label basis. Patients in the WL group did not receive any interventions including a placebo to treat their CRF during this phase. Starting day 8, patients in both WL and OLP groups received 1 tablet of OLP twice a day for 3 weeks (waitlist crossover phase). Placebo was prepared from inactive excipient methylcellulose and had little or no odor or distinct taste.

Following screening and provision of informed consent, randomization was conducted using the institutional clinical trial conduct (CTC) website into 1 of 2 arms (1:1). Patients were stratified based on baseline fatigue severity score of ≤7 or >7 (severe versus moderate fatigue). After completing the study assessments at baseline (see “Outcome Measures” section below), the investigators (SY or AA) provided instructions regards to the study intervention as per the standardized script (Supplementary Material). The script was adapted verbatim from a prior published OLP study on CRF in cancer survivors.^21^ This standardized script was essential so as to have a similar reliable and consistent placebo effect as seen in this previous OLP fatigue study.^21^ Using the script, the following points were emphasized: (1) the placebo effect can be effective, (2) the body automatically can respond to taking placebo pills like Pavlov dogs who salivated when they heard a bell (condition), (3) a positive attitude can be helpful but is not necessary, and (4) taking the pills faithfully for the 7 days is critical (during the randomized controlled phase).^14-21^ The investigators were blinded to the study assignments during the process of providing the instructions. After consent, enrollment, and instructions patients were informed of the assigned group.

### Assessments

Patients’ demographic data, including age, sex, ethnicity, cancer diagnosis, and treatment history, were recorded at the time of randomization

### Outcome Measures

The patients’ completed FACT-G,^26^ FACIT-F,^27^ ESAS,^25^ MFSI-SF,^28,29^ and CES-D,^30^ at baseline, days 8, 15, and 29; Godin^31^at baseline, Days 8, 29; and GSE^32^ at Days 8 and 29.

FACT-G and FACIT-F are part of the collection of health-related quality-of-life questionnaires targeted at the management of chronic illness referred to as **the** FACIT Measurement System.^26,27^ FACT-G is a quality-of-life (QOL) instrument widely used for in cancer clinical trials.^26,27^ It consists of 27 general quality-of-life questions divided into 4 domains (physical, social, emotional, and functional). All items in the FACT-G scale use a 5-point rating scale (0 = not at all; 1 = a little bit; 2 = somewhat; 3 = quite a bit; and 4 = very much). The FACT-G total score is computed as the sum of the 4 domains. The reliability and validity of FACT-G in cancer patients are well established.^26^

FACIT-F is a 13-item scale commonly used to assess CRF. FACIT-F measures individuals’ level of fatigue during their usual daily activities over the past 1 week.^27^ Similar to FACT-G it is a 5-point rating scale with recall for each item being 7 days. The calculated score for FACIT-F varies from 0 to 52.^27^ For FACT-G and FACIT-F, the higher the score the better the quality of life, or fatigue, respectively. FACIT-F has a sensitivity of 0.92, specificity of 0.69, internal validity (Cronbach *α* >0.93-0.95), and test-retest reliability (*R* = 0.90).^27^

ESAS was used to assess 10 symptoms commonly experienced by cancer patients during the previous 24 h: pain, fatigue, nausea, depression, anxiety, drowsiness, dyspnea, anorexia, sleep, and feelings of well-being.^25^It is a 0-10 scale, with 0 = no symptom, 10 = worst possible. For the purpose of “post hoc” analysis,^31,33-35^ we defined the ESAS physical distress score as the sum of pain, shortness of breath, appetite, nausea, fatigue, and drowsiness scores; ESAS psychological distress score, sum of anxiety, and depression scores. The fatigue cluster was defined as a composite of ESAS fatigue, pain and depression items. These definitions of fatigue cluster, ESAS physical, and ESAS psychological distress scores were based on prior studies in cancer patients as these symptoms often co-occur and share a common pathobiology (increase in inflammatory cytokines).^33-38^

MFSI-SF is a 30-item scale and was used to assess the multidimensional nature of fatigue, and fatigue disrupted QOL.^28^ Responses for each item during the past week are made using a 5-point scale, ranging from 0 = not at all to 4 = extremely. Ratings were summed to obtain 5 subscales: general, physical, emotional, mental, and vigor, with a higher score indicating higher levels of fatigue experienced by the patient. The MFSI-SF and its subscales are validated with Cronbach *α* of 0.83-0.92.^29^

The primary outcome (FACIT-F) of our study, is a highly validated tool that our team and others have previously used in multiple CRF treatment trials.^4,6,8-11,39^ In addition, our goal in this study was also to determine the sensitivity to change of OLP to a number of CRF tools (MFSI-SF, and ESAS—fatigue item) that might be of interest for future fatigue research.

*CES-D* is a brief self-report scale designed to measure self-reported symptoms associated with depression experienced in the past week.^30^ The scale includes 20 items comprising 6 subscales reflecting major facets of depression: depressed mood, feelings of guilt and worthlessness, feelings of helplessness and hopelessness, psychomotor retardation, loss of appetite, and sleep disturbance. Patients rate the duration 0-3 range for each item (0 = rarely or none of the time, 1 = some or little of the time, 2 = moderately or much of the time, 3 = most or almost all the time). The global score is the sum of these 20 symptoms with high scores indicating more severe depressive symptoms. The scale has been found reliable (*α* > 0.85) in the previous research.^30^

Using the *GODIN* questionnaire participants were asked how many times per week on average they participated in strenuous, moderate, and mild exercises for more than 15 min during their free time.^31^

*GSE:* On days 8 and 29, we assessed the patient-reported perception of change in their fatigue levels (worse, about the same, or better) after starting the intervention. If the patient answers “better,” we further asked the patient to select how much better: “almost the same, hardly better at all, a little better, somewhat better, moderately better, a good deal better, a great deal better, or a very great deal better.” If the patient answered “worse,” we further asked them to select how much worse: “almost the same, hardly any worse at all, a little worse, somewhat worse, moderately worse, a good deal worse, a great deal worse, or a very great deal worse.^32^”

Toxicity assessment in the treatment groups was assessed by the research coordinator using Common Terminology Criteria for Adverse Events (CTCAE) Version 5. The research coordinator was not blinded to the patient study assignment during the assessment of toxicity assessment.

### Statistical Analysis

Demographic, clinical characteristics, and baseline scores were summarized by treatment group using summary statistics. Adherence was calculated as a median (IQR) prescribed study medication on day 8 in the OLP group, and on day 29 in each of the 2 groups.

For the primary FACIT-F analyses, the change scores were calculated for each group, and the difference in these 2 scores was examined with 2-sided 2 sample *t*-tests.

Similarly, for the secondary outcomes including FACT-G subscales, and total score, MFSI-SF, ESAS fatigue, ESAS symptom distress scores, ESAS fatigue/pain/depression cluster, CES global, GODIN strenuous exercise, GODIN moderate exercise, GODIN mild exercise scores, the change scores were calculated for each group at each post-baseline time point (days 8, 15, and 29), and the difference in these scores were examined with 2-sided 2 sample t-tests.

For GSE, we summarized by treatment groups independently at each time point (days 8 and 29), with differences assessed by chi-square tests. Supplementary mixed-effect models were used but not reported (for the reason of simplicity, and per the pre-specified protocol) which included contrast estimates following group * time interaction, and these results were consistent with the estimates from *t*-tests, and helped to reassure us that the simple summaries and *t*-tests were not substantially biased by baseline differences or post-baseline missing data.

Counts per subject of adverse events during the study were summarized by treatment using summary statistics. The difference in counts between treatment groups was assessed by a negative binomial analysis of the variance model. In addition, the maximum grade per subject of adverse events (with grades 1-2 and 3-5 combined) was summarized by treatment as counts and percentages by grade, with the overall difference assessed by Fisher’s exact test. A 95% level of statistical significance was assumed in all statistical testing. Statistical analyses were performed using R statistical software (R Core Team, 2020, version 3.6.3).^40^

Sample size calculation: The primary outcome in this study was the change in FACIT-F score on day 8 between the OLP and WL groups. Based on prior placebo studies by our team,^23,24^ an *a priori* power analysis suggested that, with 41 patients per treatment group, we would be able to detect a change in FACIT-F (standard deviation of 9.0) of at least 5.59 with 80% power at the .05 significance level.

## Results

**Figure 1.**
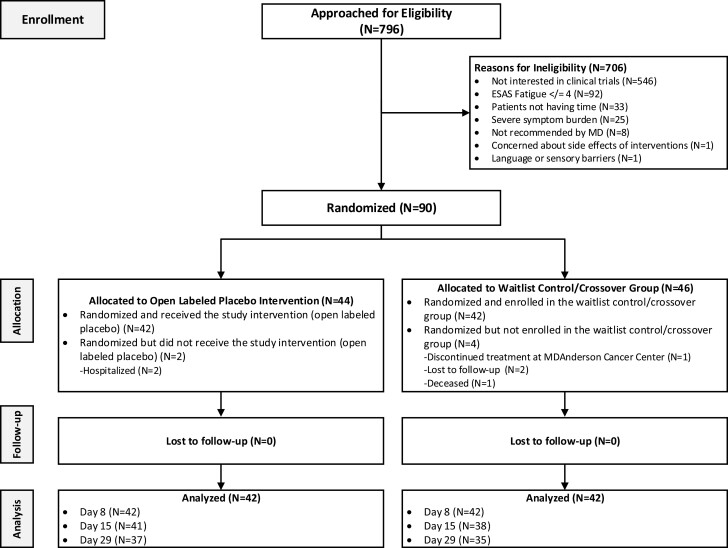
CONSORT diagram: details of enrollment, allocation, follow-up, and analysis.

A total of 84 out of 90 (93%) patients were evaluable. The reason for dropout in the OLP group was hospitalization (*n* = 2); reasons for dropout for the WL group were: (a) patient discontinued treatment at MD Anderson Cancer Center (*n* = 1), (b) lost to follow-up (*n* = 2), and (c) deceased (*n* = 1) (Fig. 1), consort diagram] demographic, clinical characteristics, and baseline scores (Table 1) showed no significant differences between groups in age, gender, ethnicity, education, treatment, and baseline assessment scores.

**Table 1.** Patient demographics and clinical characteristics at baseline.

Characteristics	OLP group, *n* = 42	WL group, *n* = 42	Total, *N* = 84	*P* ^a^
Age, years, mean (SD)	57 (12)	55 (14)	56 (13)	.50
Sex, *n* (%)				
Female	31 (74)	25 (60)	56 (67)	.25
Race, *n* (%)				
Caucasian	33 (79)	28 (67)	61 (73)	.68
African American	5 (12)	6 (14)	11 (13)	
Asian	1 (2)	1 (2)	2 (2)	
Other	3 (7)	7 (17)	10 (12)	
Marital status, *n* (%)				
Married	27 (64)	31 (74)	58 (69)	.74
Single/never married	6 (14)	5 (12)	11 (13)	
Divorced	5 (12)	2 (5)	7 (8)	
Single/lives with partner	3 (7)	2 (5)	5 (6)	
Widowed	1 (2)	2(5)	3(4)	
Employment status, *n* (%)				
Full-time	15 (36)	13 (31)	28 (33)	.80
Retired	16 (38)	19 (45)	35 (42)	
Unemployed	6 (14)	7 (17)	13 (15)	
Others^b^	5 (12)	3 (7)	8 (10)	
Highest education level, *n* (%)				
Less than high school	0 (0)	3 (7)	3 (4)	.41
High school/tech school	13 (31)	12 (29)	25 (30)	
College or higher	24 (57)	21 (50)	45 (54)	
Not reported	5 (12)	6 (14)	11 (13)	
Diagnosis, *n* (%)				
Breast	15 (36)	14 (33)	29 (35)	.85
Thoracic	9 (21)	7 (17)	16 (19)	
Gastrointestinal/colon	5 (12)	9 (21)	14 (17)	
Genitourinary	3 (7)	5 (12)	8 (10)	
Gynecological	3 (7)	3 (7)	6 (7)	
Sarcoma	1 (2)	2 (5)	3 (4)	
Melanoma	1 (2)	1 (2)	2 (2)	
Others	5 (12)	1 (2)	3 (4)	
Type of anti-cancer therapy, *n* (%)				
Radiation and chemotherapy	31(74%)	34(81%)	65 (77%)	.58
No cancer treatment	7 (17%)	6 (14%)	13 (15%)	
Chemotherapy	2 (5%)	2 (5%)	4 (5%)	
Target therapy	2 (5%)	0 (0%)	2 (2%)	
Subjective and objective measures, mean (SD)				
FACIT fatigue	24.3 (8.9)	26.1 (10.0)	25.2 (9.5)	.38
FACT-G total	59.7 (7.2)	58.6 (9.1)	59.2 (8.2)	.55
Physical wellbeing	11.4 (4.5)	11.4 (4.8)	11.4 (4.6)	.98
Social/family wellbeing	23.1 (5.2)	23.6 (5.8)	23.3 (5.5)	.66
Emotional wellbeing	9.0 (4.0)	7.5 (3.3)	8.3 (3.8)	.07
Functional wellbeing	16.2 (5.7)	16.0 (5.3)	16.1 (5.4)	.85
Physical wellbeing	11.4 (4.5)	11.4 (4.8)	11.4 (4.6)	.98
ESAS pain	3.5 (2.3)	4.1 (3.1)	3.8 (2.7)	.31
ESAS fatigue	6.1 (2.0)	5.9 (1.8)	6.0 (1.9)	.56
ESAS fatigue cluster	1.1 (0.5)	1.2 (0.5)	1.1 (.5)	.64
ESAS physical distress	20.3 (9.1)	20.6 (9.9)	20.5 (9.5)	.90
ESAS psychological distress	4.2 (4.6)	4.4 (3.4)	4.3 (4.0)	.84
MFSI-SF global	21.0 (13.8)	19.6 (12.5)	20.3 (13.1)	.61
General fatigue	14.0 (5.0)	13.5 (4.6)	13.7 (4.8)	.62
Physical fatigue	7.2 (4.9)	7.2 (4.6)	7.2 (4.7)	1.00
Emotional fatigue	6.3 (5.8)	5.7 (4.5)	6.0 (5.2)	.62
Mental fatigue	7.1 (5.3)	6.3 (4.2)	6.7 (4.8)	.43
Vigor	13.6 (3.4)	13.1 (3.4)	13.3 (3.4)	.52
CESGlobal	35.9 (9.9)	34.0 (6.5)	34.9 (8.4)	.29
GODIN strenuous exercise	0.1 (0.6)	0.1 (0.6)	0.1 (.6)	1.00
GODIN moderate exercise	1.4 (2.6)	0.6 (1.2)	1.0 (2.1)	.09
GODIN mild exercise	3.7 (2.8)	3.4 (2.6)	3.6 (2.7)	.58

Chi-square/Fisher’s exact test for categorical variables and independent *t*-test for continuous variables.

Others: homemaker, part-time, disability.

Abbreviations: CES, The Center for Epidemiologic Studies—Depression; Godin, Godin Leisure Time Exercise Questionnaire. ESAS Physical Distress, sum scores of ESAS items: Pain, Fatigue, Nausea, Drowsiness, Dyspnea and Appetite; ESAS Psychological Distress, sum scores of ESAS items: Depression and Anxiety; ESAS, Edmonton Symptom Assessment Scale; Fatigue Cluster: sum scores of ESAS items: pain, fatigue, depression; FACIT-F, Functional Assessment of Chronic Illness Therapy-Fatigue; FACT-G, Functional Assessment of Cancer Therapy-General; HADS, Hospital Anxiety Depression Scale; MFSI-SF, Multidimensional Fatigue Symptom Inventory-Short Form; OLP Group, Open-labeled placebo group; WL Group, Waitlist control/cross over group group.

The median % (interquartile range) adherence to the study medication was 100% (100, 100), and 97.3% (86.6, 100) on days 8 and 29, respectively in the OLP group, and 100% (95.2, 100) on day 29 in the WL group. No statistical differences in study medication adherence were found on day 29 between the 2 groups (*P* = .12).

**Figure 2.**
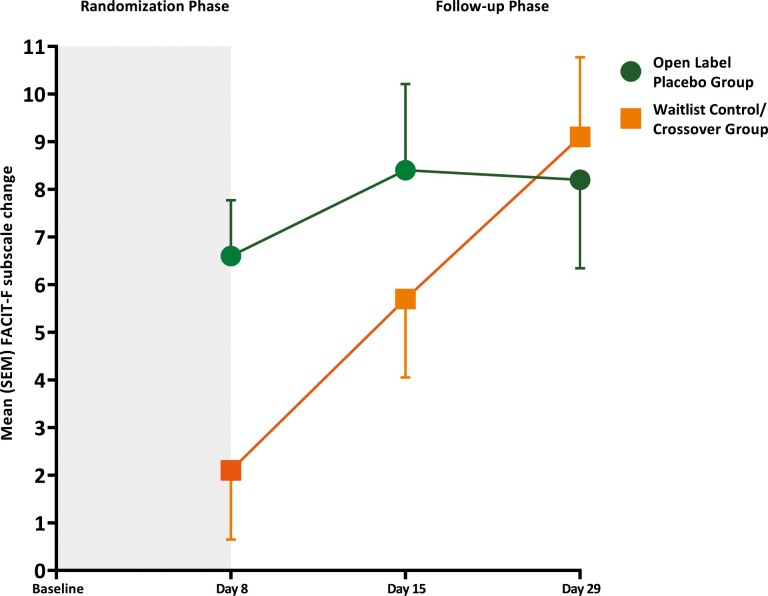
FACIT-F by treatment and time point, shows change in fatigue scores on day 8, D15, and D29 in open-label placebo group and waitlist control/cross over group. In the follow-up phase both arms received open-labeled placebo for 3 weeks.

The change from baseline in FACIT-F score at day 8 was significantly better (*P* = .016) in the OLP group with a mean difference of 4.6 units as compared to the WL group (Table 2, Fig. 2). The effect size was 0.70. We found no differences between both the groups regards to change from baseline in FACIT-F scores on days 15, and 29, when the WL group also received a placebo.

**Table 2.** Changes in fatigue subjective and objective measures at days 8, 15, and 29 from baseline in open-labeled placebo and waitlist control/cross over groups.

Main outcomes, mean (SD)	Randomized control phase^a^			Follow-up phase^a^					
	Change from baseline to day 8			Change from baseline to day 15			Change from baseline to day 29 (*N* = 37)		
	OLP arm (*N* = 42)	WL arm (*N* = 42)	*P* ^b^	OLP arm (*N* = 41)	WL arm (*N* = 38)	*P* ^b^	OLP arm (*N* = 37)	WL arm (*N* = 35)	*P* ^b^
FACIT fatigue	6.6 (7.6)	2.1 (9.4)	**.016**	8.4 (11.6)	5.7 (9.9)	.28	8.2 (11.3)	9.1 (9.9)	.73
FACT-G total	−4.4 (5.3)	1.0 (7.9)	**.002**	−4.0 (5.0)	−2.3 (6.8)	.28	−5.9 (11.0)	−6.9 (16.2)	.78
Physical wellbeing	−2.3 (3.6)	−1.1 (4.3)	.23	−2.9 (4.8)	−2.6 (5.1)	.83	−3.1 (5.9)	−2.5 (6.5)	.71
Social wellbeing	−0.5 (3.1)	0.6 (4.0)	.20	−0.6 (3.2)	−0.7 (3.8)	.91	0.0 (4.8)	−1.6 (6.3)	.28
Emotional wellbeing	−1.4 (3.0)	0.2 (3.0)	**.030**	−2.0 (4.5)	−0.6 (2.7)	.13	−2.2 (5.6)	−0.4 (4.0)	.15
Functional wellbeing	−0.2 (4.2)	1.0 (3.7)	.18	1.4 (4.6)	1.4 (4.3)	1.00	2.5 (8.0)	3.5 (11.2)	.71
ESAS fatigue	−1.5 (1.9)	−0.6 (1.9)	**.029**	−1.6 (3.1)	−1.7 (2.1)	.88	−2.4 (2.8)	−2.5 (2.5)	.92
ESAS fatigue cluster	−0.3 (0.4)	−0.1(0.5)	**.044**	−0.3(0.6)	−0.3(0.4)	.56	−0.4(0.6)	−0.4(0.6)	.95
ESAS physical distress	−5.3 (7.1)	−2.8 (7.7)	.16	−5.3 (9.3)	−4.8 (6.7)	.79	−6.4 (10.3)	−5.5 (8.0)	.71
ESAS psychological distress	−1.3 (2.8)	−0.8 (3.1)	.49	−1.6 (3.9)	−1.4 (3.0)	.80	−2.2 (4.3)	−1.0 (3.6)	.25
MFSI−SF global	−7.6 (11.8)	−3.4 (13.0)	.16	−12.4 (15.7)	−8.8 (12.9)	.34	−12.3 (15.6)	−12.0 (14.5)	.95
General fatigue	−3.0 (5.3)	−1.7 (5.5)	.32	−5.1 (7.6)	−3.8 (6.6)	.47	−5.9 (6.9)	−5.1 (6.8)	.63
Physical fatigue	−0.9 (4.2)	−1.1 (3.8)	.79	−2.3 (3.9)	−2.0 (3.6)	.78	−2.2 (5.0)	−2.8 (4.9)	.63
Emotional fatigue	−2.0 (4.2)	0.0 (4.5)	.06	−3.0 (5.5)	−1.3 (4.1)	.19	−2.8 (6.0)	−2.3 (4.8)	.72
Mental fatigue	−2.1(4.6)	−0.5 (4.7)	.15	−3.1 (4.6)	−1.5 (3.0)	.11	−2.9 (4.9)	−2.9 (3.6)	.97
Vigor	−0.3(2.6)	0.2(2.9)	.48	−1.1 (2.9)	0.2 (3.7)	.15	−1.6 (3.8)	−1.0 (4.0)	.59
pCES global	−2.6 (6.5)	−1.1 (5.3)	.26	−4.8 (9.0)	−2.2 (7.7)	.21	−5.6 (10.0)	−2.7 (8.7)	.23

In the randomized control Phase, OLP group received open-labeled placebo for 1 week, and WLC group were in waitlist for 1 week; After 1 week, both OLP group and WLC group received open-labeled placebo.

Independent *T*-test.

Abbreviations: CES, The Center for Epidemiologic Studies–Depression; ESAS Physical Distress Score, sum scores of ESAS items: Pain, Fatigue, Nausea, Drowsiness, Dyspnea and Appetite; ESAS Psychological Distress Score, sum scores of ESAS items: Depression and Anxiety; ESAS, Edmonton Symptom Assessment Scale; FACIT-F, Functional Assessment of Chronic Illness Therapy-Fatigue; FACT-G, Functional Assessment of Cancer Therapy-General; Fatigue Cluster: sum scores of ESAS items: pain, fatigue, depression; GSE, Global Symptom Evaluation. HADS, Hospital Anxiety Depression Scale; MFSI-SF, Multidimensional Fatigue Symptom Inventory-Short Form; OLP Group, open-labeled placebo group; WL Group, waitlist control group/cross over group.

Bolded text suggests that the P value was statistically significant.

Among secondary outcomes, ESAS fatigue, and ESAS fatigue cluster (fatigue/pain/depression) showed significant evidence of reduced CRF in the OLP group on day 8 of the study (*P* = .029, and *P* = .044, respectively) (Table 2). FACT-G total and FACT emotional well-being scores were significantly better in the WL group (*P* = .002, and *P* = .03, respectively) on day 8. There was no significant difference between groups in the MFSI-SF, CES-D, and ESAS distress scores in change from baseline scores on days 8, 15, and 29 (Table 2). There were also no significant differences between OLP and WL groups in the change from baseline on day 8, and day 29 Godin scores for mild activity (*P* = .68), moderate activity (*P = .*51), strenuous activity, *P = .*32. Supplementary Table shows the GSE responses on days 8 and 29 in OLP and WL Groups.

There was no significant evidence of a difference between groups in counts per subject of adverse events (*P = .*29), per the negative binomial model; both OLP and WL groups had medians of 0, with corresponding interquartile radii (IQR) of 1.8 and 2.0, respectively. There was no significant difference in the type and frequency of maximum adverse events between the 2 groups, *P* = .71 (Table 3). The frequency of severe adverse events (grades 3-5) in the OLP group was 5 (pain, *n* = 3, and fatigue, *n* = 2). For the WLC group the frequency of severe adverse events were 4 (pain, *n* = 1, confusion/delirium, *n* = 2, and dizziness, *n* = 1).

**Table 3.** Adverse events by maximum grade in groups open-labeled placebo, and waitlist control/cross over groups.

Maximum adverse event grade, *N*	OLP group	WL group	*P* ^a^
1-2	9 (64%)	14 (74%)	.71
3-5	5 (36%)	5 (26%)	
Total	14	19	

Fisher’s exact test. Grading of adverse events as per National Cancer Institute Common Toxicity Criteria (version 5.0). All adverse events were unrelated to the study drug.

Abbreviations: OLP, open-label placebo group; WL, waitlist control/cross over group.

## Discussion

In this study, we found that OLP significantly improved CRF (FACIT-F) in patients with advanced cancer. The ESAS fatigue item, and fatigue cluster, showed a significant improvement in the OLP group. After week 1, when all patients received OLP, there was a similar improvement in CRF in both groups.

The most interesting aspect of our study was that we found improvement in CRF with the administration of a placebo given on an open-label basis in patients with advanced cancer. These results were consistent with prior published studies using OLP for the treatment of CRF in cancer survivors.^20,21^ Our study differed from the prior 2 OLP studies in that the primary outcome was measured earlier (on day 8), and was limited to patients with advanced cancer.^20,21^ The improvement in CRF was similar (effect size of.70) to these prior studies.^20,21^ Additionally, we found improvement of CRF after 4 weeks of treatment of OLP suggesting sustained improvement with OLP.

As seen in prior OLP studies,^12-21^our study found that there was good adherence to the use of the placebo medication which was administered in an open-label basis which suggests an acceptability of the use of placebo medication. The improvement in CRF with OLP and adherence suggests the overall beneficial effects of the placebo for the treatment of CRF in advanced cancer.

In our study, QOL [FACT-G scores] did not improve in the OLP group. Fatigue disrupted QOL (MSFI-SF global score) was also not significantly better in the OLP group than in the WL group. Similarly, we did not find significant improvement in the secondary outcomes such as CES-D and Godin scores. The placebo message was given exclusively regards to the symptom of fatigue and no other symptoms that are part of the QOL construct such as pain, depression, anxiety, function status, and this lack of improvement in QOL and other secondary outcomes suggests specificity of the placebo expectation of improvement.^41^Another explanation is that these secondary outcomes were exploratory and therefore not adequately powered.

Similarly, the significant improvement in the ESAS fatigue item and the fatigue cluster with no improvement seen in ESAS symptom distress scores may also be related to the effects of the OLP being focused upon on the subjective symptom of fatigue rather than the symptom distress scores. Another possible explanation is that the ESAS symptom distress and FACT-G scores are unlikely to be driven by fatigue alone and that perhaps no fatigue treatment would be likely to address them all to a large extent. Further, well-powered studies are needed to validate the findings of these important secondary outcomes.

The amount of improvement in CRF seen in the patients who received OLP group (6.6 and 8.2 on day 8, and day 29) was greater than the minimally clinically important difference (MCID) for FACIT-F,^42,43^ and this was also better than the improvement of CRF scores seen in most of the prior clinical trials for the treatment of CRF using pharmacological interventions such as erythropoietin and methylphenidate.^7,43^ In addition, the improvement in CRF was maintained for at least until day 29 (treatment duration of OLP). These findings emphasize the potential benefit of OLP in the clinical management of CRF in patients with advanced cancer. It also suggests that instead of using OLP as a control, future CRF treatment trials should consider using a combination of OLP with other efficacious CRF treatments (eg, methylphenidate or physical activity) so as to take advantage of this important placebo response. This combination of pharmacological intervention to treat CRF (eg, methylphenidate) with OLP interventions might have a synergistic effect in improving subjective response to CRF, and perhaps maybe allowing for dose reduction, and therefore a reduction in toxicity of the pharmacological intervention used to treat CRF.

In clinical practice, patients with advanced cancer might be offered to use OLP to treat CRF immediately as they are being investigated for possible causes of CRF. OLP may improve CRF by building an effective patient-physician relationship via trust and respect.^12-21,44^ Further studies are needed prior to integration into clinical practice.

Future studies should evaluate how long the effects of the placebo last after discontinuing placebo medication in patients with advanced cancer. This information is very useful for designing CRF pharmacological trials in patients with advanced cancer which often use a placebo as a control. Further studies are also needed to understand the mechanisms by which the placebo improves CRF in advanced cancer.^45^

Our study has several limitations. The generalizability of the study results may be somewhat limited as the study was conducted in a single tertiary cancer center. The design of the study may have potentially added performance and obsequiousness bias.^46^ One of the limitations o our study was that the toxicity assessment was performed by the study coordinator who was not blinded to the study assignment. However, while this might be a limitation of our study patients reported a low frequency of adverse events in both study groups. Another limitation of the study was that we have not made any corrections for multiple statistical tests despite a large number of outcomes, however since the outcomes should be highly correlated (since they all are measuring some aspect of fatigue), typical adjustments for multiple testing would be excessively conservative. Finally, this study was not adequately powered to determine the efficacy of OLP on secondary outcomes and adverse reactions. Further studies are needed.

## Conclusion

Open-labeled placebo (OLP) was efficacious in reducing CRF as measured by the FACIT-F, ESAS fatigue item, and fatigue cluster scores in fatigued advanced cancer patients at the end of 1 week. The improvement in fatigue was maintained for 4 weeks of the study period. Further studies of this intervention are justified.

## Supplementary Material

oyac184_suppl_Supplementary_Material

oyac184_suppl_Supplementary_Table

## Data Availability

The data underlying this article will be shared upon reasonable request to the corresponding author.
