# Supplementary material for: Open-Label Placebo for the Treatment of Cancer-Related Fatigue in Patients with Advanced Cancer: A Randomized Controlled Trial
Source: Oncologist. 2022 Sep 15;27(12):1081–9. doi: 10.1093/oncolo/oyac184 (PMC9732231; doi:10.1093/oncolo/oyac184)
Supplement: oyac184_suppl_Supplementary_Material [file oyac184_suppl_supplementary_material.docx]

**Supplementary material**

This script for the instructions provided by the investigators was adapted from prior published open label placebo study by Zhou et al.^21^ This standardized script was essential so as to have a similar reliable and consistent placebo effect as seen in this study.

This adaptation has been reprinted with the permission from Springer Nature, Open-label placebo reduces fatigue in cancer survivors: a randomized trial. Author: Eric S. Zhou et al; Publication: Journal of Supportive Care in Cancer; Date: Oct 10, 2018.

**A. Study Script by the treating Clinician in all patients enrolled in the study:**

1. **Introduction**

- Cancer related fatigue in patients with advanced cancer is a subjective sense of tiredness or exhaustion not relieved with rest and interferes with daily function.
- It can interfere with your quality of life, and ability to receive cancer treatment consistently.
- To date there is no effective medication to treat cancer related fatigue in patients with advanced cancer.
- I would like to discuss the placebo effect and use of Placebo as a part of clinical trial to relieve your fatigue.
- [ pause], Now elicit understanding by terms such as “Does it sound OK?”]
- Ask the patient if they have any questions regards to participation in this study, or consent. Use terms which include “Do you have any questions about participation in the study? Or Consent? If the response is positive regards to complete understanding you may proceed to next step.
- For you to participate I want to give you some more information about the placebo effect, and then I will give you instructions about taking the placebo to relieve your fatigue and completing the study measures. Does that sound ok?

1. **Background on Placebo**

**What is a placebo effect?** Placebos do not contain any active medication, and yet we know that they can have a powerful ability to improve symptoms for many people across a wide range of diseases. When it comes to fatigue in patients with advanced cancer, several studies have been done by our team and others to see if stimulant medications or nutraceuticals such as Ginseng can improve fatigue in cancer patients. In these studies, one group of patients received the stimulant medication, and the other received a placebo. All of these studies have shown that patients who received the placebo showed significant improvement in their fatigue—they even improved as much as the patients who received the stimulant medications or Ginseng.

**How does placebo work?** This placebo effect may seem surprising, but we think it can be an important way to help cancer patients improve their fatigue symptoms as it can harness the **power of your mind-body connection**. We believe that one way placebo may work is automatically through a mind-body mechanism called **conditioning**.

**What is conditioning?** Across their lifetimes, most people have been successfully treated with a variety of medications. This consistent experience of positive improvement after taking a medication automatically conditions the person’s body to self-heal in response to taking a pill. Their body expects to feel better after taking a medication, and this anticipation may lead to actual physical changes. So when a person takes a medication, in addition to the improvement in symptoms that occurs due to the medication, people also experience improvement due to this automatic self-healing process. Do you have any questions so far? *(Pause to allow for* *participant comment or response)*

**III. Use of a Placebo in the Study**

- In previous studies to test the benefit of various medication to relieve fatigue the patients were not aware whether they were receiving the study medication or placebo, yet they showed benefit from placebo in relieving fatigue. Recent studies in various symptoms including cancer related fatigue in cancer survivors, low back pain, migraine headaches, irritable bowel symptoms found that the use of placebo without concealment, that is the patient know they are taking placebo. The improvements were larger in some studies without than with concealment. Therefore, in this study we are interested in finding out if participants experience improvement in their fatigue symptoms – even when they know they are taking placebos.
- So, for this study, we will ask you to take placebo pills for your fatigue every day for one week.
- Research studies have also shown that the more faithfully and regularly a person takes a placebo, the better the outcome. The idea is that each time a person takes a pill, the automatic mind-body, self-healing process may be activated. So, to receive the maximum benefit, it’s important to take the pills exactly as you are asked to. We ask that you take one pill twice a day, preferably when you wake up and when you go to bed. Some people find it more convenient to take them around meal times, and that is another alternative, like 1 pills at breakfast and 1 pills at dinnertime.
- [ pause], Now elicit understanding by terms such as “Does it sound OK?”]
- Some people may have questions about whether or not a placebo will work for them, and that is ok. We think that just taking the pills will have a positive effect on people. At the same time, we also think that positive expectations can improve people’s symptoms. So the placebo pill may have a greater effect if you keep in mind that placebos have already been shown in other studies to have a powerful and effective in their ability to reduce symptoms such as of fatigue.
- I also wanted to remind you that everyone in the study will get a chance to try the placebo treatment but half the participants will start the placebo right away and the other half will be asked to wait for 1 week before starting the placebo. The reason for that is it is very important for us to know how fatigue symptoms change for people while taking the placebo compared to those not taking the placebo. We can’t do the study unless people are willing to wait, and we really appreciate that you are willing to join the study now, even though you know you may be in the group that has to wait for one week. At the end of our meeting today, we will tell us which group you will be in. This would be done by randomization (flip of coin) performed by the computer. Any questions for me at this point? *(Pause* *to allow for participant comment or response)*

**IV. Group Assignment and Final Instructions:**

Placebo Participants Only: You are in the upfront placebo group which means that we will ask you to start the placebo today by taking 1 pill tonight when you go to bed, or at your next meal, and then start taking 1 pill twice a day. We will be giving you a call in 7 days to see how things are going, and then again in about 3 weeks to check in with you and ask you to complete the final measures for the study. These phone calls will be brief, about 15 minutes, and you will need to have your forms with you during the phone calls.

Let me show you the forms you will need to complete and mail back to us. Any questions?

No Treatment Control Participants Only: You are in the one week waiting period group which means that we will ask you to stay in touch with us for the next week and then we will send you the placebos in the mail for you to try. We will be giving you a call in 7 days to see how things are going, and then again in about 3 weeks from then to check in with you and ask you to complete the final measures for the study. These phone calls will be brief (about 15 minutes), and you will need to have your forms with you during the phone calls. Let me show you the forms you will need to complete and mail back to us. Any questions?

**V. Additional material that may be used in response to participant comments or Questions**

**Scenario 1**: [If participants talk about it feeling strange or silly to take a placebo]

Yes, I think that many people may feel the way you do, that it will be [silly/strange/their phrase here] to take a placebo, but it is important to remember that many people have felt better after taking placebos, and that in cancer patients with fatigue, placebos have consistently had powerful effects. We don’t know exactly how each person will respond but, we do think it is very possible that people will feel benefits from the placebo. It is helpful to keep an open mind, and remember that these placebo activations can happen independent of what you believe or think. The placebo pill is likely to have a greater effect if you keep in mind each time that you take the pill, that placebos have already been shown to have a powerful ability to reduce symptoms of fatigue.

**Scenario 2**: [If person comments on feeling uncomfortable that effects are “all in their head”]

I know what you mean—It can be hard to understand how a placebo works if there is no

medication inside, and people can wonder if the effects are “all in their head.” There is a lot we don’t understand about the placebo effect, and that is why we are doing studies like this one you are joining, but we do believe that placebos can work automatically through a mind-body mechanism called conditioning. As I mentioned, most people have been successfully treated with a variety of medications in the past, and this experience of positive improvement after taking a medication automatically conditions the person’s body to self-heal in response to taking a pill. So even though the idea of the pill is “in their head” it may have actual effects on their body. Their body expects to feel better after taking a medication, and this anticipation may lead to actual physical changes. We don’t know for sure, of course and that is why we are doing this study, but we do think there is reason to believe that the placebo can have a real effect on people’s physical functioning and on their symptoms.
