# Supplementary material for: Open-Label Placebo for the Treatment of Cancer-Related Fatigue in Patients with Advanced Cancer: A Randomized Controlled Trial
Source: Oncologist. 2022 Sep 15;27(12):1081–9. doi: 10.1093/oncolo/oyac184 (PMC9732231; doi:10.1093/oncolo/oyac184)
Supplement: oyac184_suppl_Supplementary_Table [file oyac184_suppl_supplementary_table.docx]

**Appendix: Supplementary Table**

| **Online Table 1. Global Symptom Evaluation at Day 8 and Day 29 in in Open labeled placebo and Waitlist Control/Cross over Groups** | | | | | | |
| --- | --- | --- | --- | --- | --- | --- |
| **Global Symptom Evaluation, N (%)** | **Day 8** | | | **Day 29** | | |
|  | **OLP group** | **WL group** | **P*** | **OLP group** | **WL group** | **P*** |
| **How are symptoms?** | | | | | | |
| Worse | 2(5) | 3(8) | .73 | 1(3) | 1(3) | 1.00 |
| About the same | 26(68) | 26(72) |  | 12(35) | 11(35) |  |
| Better | 10(26) | 7(19) |  | 21(62) | 19(61) |  |
| **Symptom Change Level: If better…** | | | | | | |
| Almost the same, hardly any better at all | 0(0) | 0(0) | .74 | 1(5) | 0(0) | .59 |
| A little better | 3(30) | 3(43) |  | 3(14) | 5(26) |  |
| Somewhat better | 5(50) | 1(14) |  | 2(10) | 3(16) |  |
| Moderately better | 0(0) | 0(0) |  | 6(29) | 3(16) |  |
| A good deal better | 1(10) | 1(14) |  | 7(33) | 4(21) |  |
| A great deal better | 1(10) | 1(14) |  | 2(10) | 4(21) |  |
| A very great deal better | 0(0) | 1(14) |  | 0(0) | 0(0) |  |
| **Symptom Change Level: If worse…** | | | | | | |
| Almost the same, hardly any worse at all | 0(0) | 0(0) | 1.00 | 0(0) | 0(0) | 1.00 |
| A little worse | 0(0) | 0(0) |  | 0(0) | 0(0) |  |
| Somewhat worse | 1(50) | 1(33) |  | 0(0) | 1(100) |  |
| Moderately worse | 0(0) | 1(33) |  | 0(0) | 0(0) |  |
| A good deal worse | 0(0) | 1(33) |  | 0(0) | 0(0) |  |
| A great deal worse | 0(0) | 0(0) |  | 0(0) | 0(0) |  |
| A very great deal worse | 1(50) | 0(0) |  | 1(100) | 0(0) |  |
| **Abbreviation: OLP group**, Open label placebo group; WL group, Waitlist Control/Cross over group.  *****Fisher’s exact test. | | | | | | |
